# BioSharing: curated and crowd-sourced metadata standards, databases and data policies in the life sciences

**DOI:** 10.1093/database/baw075

**Published:** 2016-05-17

**Authors:** Peter McQuilton, Alejandra Gonzalez-Beltran, Philippe Rocca-Serra, Milo Thurston, Allyson Lister, Eamonn Maguire, Susanna-Assunta Sansone

**Affiliations:** ^1^Oxford e-Research Centre, University of Oxford, 7 Keble Road, Oxford OX1 3QG, UK; ^2^CERN European Laboratory for Particle Physics, Route de Meyrin, Geneva 23, 1211, CH, Switzerland

## Abstract

BioSharing (http://www.biosharing.org) is a manually curated, searchable portal of three linked registries. These resources cover standards (terminologies, formats and models, and reporting guidelines), databases, and data policies in the life sciences, broadly encompassing the biological, environmental and biomedical sciences. Launched in 2011 and built by the same core team as the successful MIBBI portal, BioSharing harnesses community curation to collate and cross-reference resources across the life sciences from around the world. BioSharing makes these resources findable and accessible (the core of the FAIR principle). Every record is designed to be interlinked, providing a detailed description not only on the resource itself, but also on its relations with other life science infrastructures. Serving a variety of stakeholders, BioSharing cultivates a growing community, to which it offers diverse benefits. It is a resource for funding bodies and journal publishers to navigate the metadata landscape of the biological sciences; an educational resource for librarians and information advisors; a publicising platform for standard and database developers/curators; and a research tool for bench and computer scientists to plan their work. BioSharing is working with an increasing number of journals and other registries, for example linking standards and databases to training material and tools. Driven by an international Advisory Board, the BioSharing user-base has grown by over 40% (by unique IP address), in the last year thanks to successful engagement with researchers, publishers, librarians, developers and other stakeholders via several routes, including a joint RDA/Force11 working group and a collaboration with the International Society for Biocuration. In this article, we describe BioSharing, with a particular focus on community-led curation.

**Database URL:** https://www.biosharing.org

## Rationale and Background

The growing movement for reproducible research and interoperability, along with an overall increase in data generation, has led to a proliferation of community-developed metadata standards, bringing with it many sociological and technological challenges (1, 2). As the costs of data production fall, the number of databases hosting that data has increased, leading to confusion over which database to use to store and search for the latest and most comprehensive data. Concurrently, there has been an increase in data standards as different community groups and databases develop different data formats, schema and reporting guidelines. This proliferation in content standards and databases creates a barrier for researchers and database maintainers, creating confusion over which standard they should use to format their data, or which database to submit their data to. There have been a number of projects that have helped bring order to this evolving field, such as the Nucleic Acid Research Database issue, this journal, and projects such as the JISC journal policy project (https://www.jisc.ac.uk/rd/projects/journal-research-data-policy-registry-pilot), which maps journal data policies with data standards and repositories. However, none of these projects connect all three aspects surrounding data deposition (i.e. the standards one should use, which particular database to deposit data, or which standards and repositories are recommended by a particular funder or journal), or answer questions such as ‘Is a particular standard mature enough to be used, which version should be used, and is it actively maintained?’ or ‘Which databases implement the most widespread and endorsed standards?’. Both standard and database maintainers can struggle in gaining visibility for their resource, to help encourage their adoption and endorsement. Conversely, librarians, data specialists, funding agencies and journal publishers often lack the resources to make an informed judgement on which database or standard to recommend to their user group. BioSharing, composed of three registries covering content standards, databases and data policies in the life sciences, aims to map the landscape of community-developed standards and databases, linking between and from them to data policies from funding agencies and journal publishers. In doing so, BioSharing aims to promote harmonisation and consistency, to reduce the reinvention and needless proliferation of standards and databases. BioSharing is a pivotal resource for the implementation of the ELIXIR-supported FAIR principles, defining the characteristics that contemporary data resources, tools and infrastructures should exhibit—to be **F**indable, **A**ccessible, **I**nteroperable and **R**eusable by third-parties (3).

BioSharing is a curated, searchable portal of linked information on content standards, databases, and (progressively) journal and funder policies in the life sciences, broadly encompassing the biological, environmental and biomedical sciences. Launched in 2011, BioSharing is an extension and natural evolution of the MIBBI portal (4) (MIBBI, Minimum Information for Biological and Biomedical Investigations; BioSharing is founded by the same core team), and is maintained as a community resource closely embedded in and co-sponsored by several infrastructure programs, including the NIH Big Data to Knowledge Initiative’s BioCADDIE (https://biocaddie.org) and CEDAR (http://med.stanford.edu/cedar.html) projects. BioSharing is also being enhanced as part of the Elixir UK node’s contribution to the ELIXIR EXCELERATE program (http://www.elixir-europe.org/excelerate/interoperability). BioSharing ensures that data standards, biological databases and data policies are registered, informative and discoverable. As a one-stop shop for standards, databases and data policies in the life sciences, BioSharing not only links these resources, but also details the relationships between them, providing context and metrics, as standards evolve and are implemented in databases, and both standards and databases are recommended by journal or funding body data policies. BioSharing also provides a historical perspective on these standards and databases, detailing when different versions of a standard are created or deprecated, and when updates to a database or policy appear, so enabling users to assess the maturity and evolution of each resource.

Although all records in BioSharing are manually curated, many have been added and edited by the community themselves, rather than BioSharing curators. Users are able to claim the record(s) for the resource(s) they maintain. This allows them to not only gain personal recognition for their work, but also ensures that the data on their resource is accurate and completely up-to-date. This community curation aspect of BioSharing, along with the linking and embedding of each record into the landscape of standards and databases, helps make BioSharing an accurate and comprehensive representation of metadata standards, databases and policies in the life sciences.

## The Three Registries

BioSharing has grown year-on-year to contain over 1300 records, split between the three registries (Standards, Databases and Policies). The Standards registry is split further into three types of data standard according to the focus: Reporting Guideline (content), Model/Format (syntax) and Terminology artifact (semantics). The Reporting Guideline subtype details the information elements that need to be expressed in order to create a common core set of descriptors for different datasets. Examples include the ARRIVE (5) (Animal Research: Reporting in-vivo experiments) guidelines, which consist of a 20-item checklist and recommendations for authors for reporting study design, experimental procedures and experimental animals. The Model/Format subtype covers data models and exchange formats that are used in data sharing. An example is the ISA-Tab format (6), which is an extensible, hierarchical structure that describes the experimental metadata with elements such as the sample characteristics, technology and measurement types used and sample-to-data relationships. Where possible, the xsd schema files for these standards are available from BioSharing, so saving time for those users who wish to see the schema directly. The third standard subtype, Terminology artifacts, are semantic representations of a topic or field used to catalogue and organise data into structural hierarchies (ontologies) with coded relationships between them (e.g. the Disease Ontology (7), that categorises human diseases, such that Parkinson’s Disease is a Neurodegenerative disease). This allows the organisation and retrieval of data in a structured manner and these standards are integral to data management and searching. This subtype is curated in collaboration with BioPortal (8) and the OBO Foundry (9).

**Figure 1:**
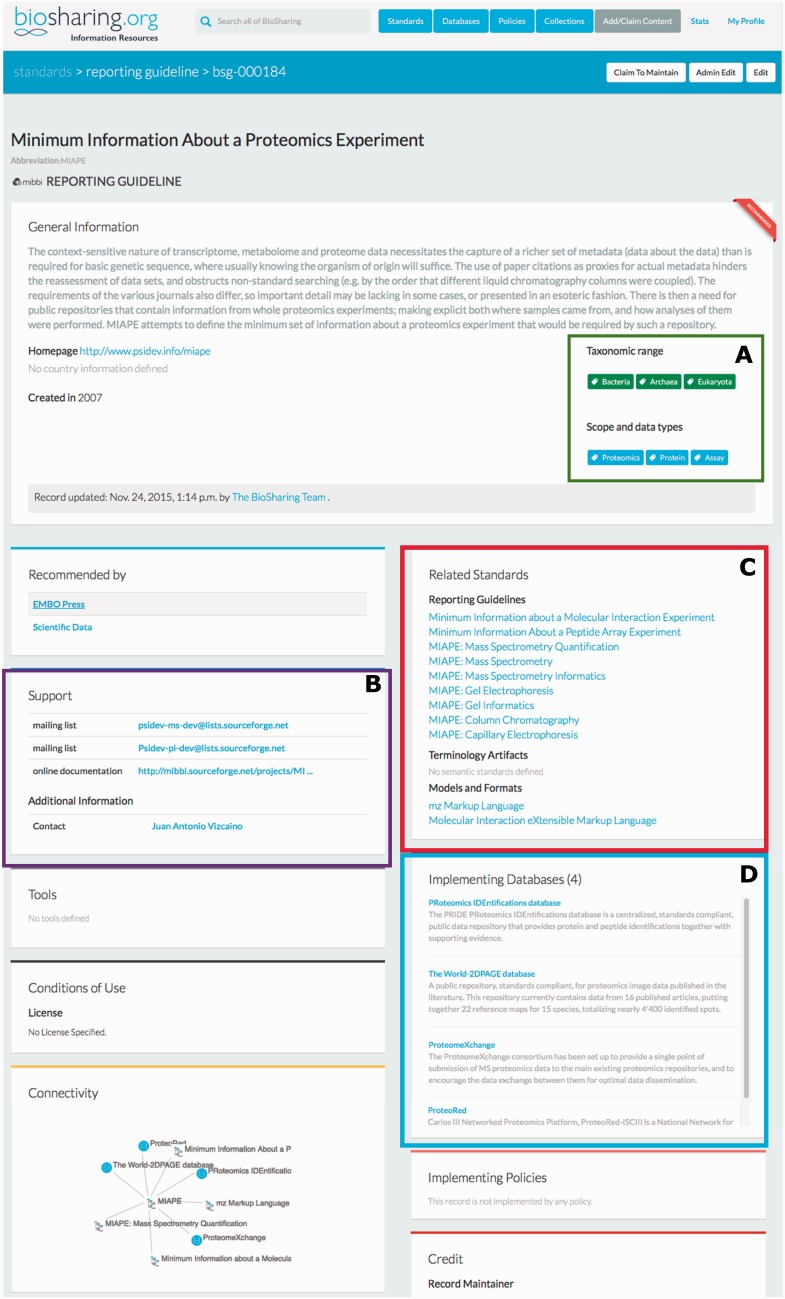
The BioSharing MIAPE Standard Record (top section). In the General Information section, the Taxonomic Range and Scope and data types tags are highlighted in a green box **(A)**. Clicking on a tag from within these two fields initiates a search for all the records annotated with that tag. The Support section **(B**—highlighted in purple) contains information on help documentation, mailing lists and contact details. The Related Standards section **(C**—highlighted in red) contains links to other metadata standards, such as the extensions to the initial MIAPE standard. Beneath this section, the implementing databases section **(D**—highlighted in blue) provides links to those resources that have implemented the MIAPE standard (e.g. The PRIDE database).

Community standards arise and iterate as the data and requirements change. BioSharing reflects this evolution by tracking the versioning, extension, and deprecation of standards. The Proteomics Standards Initiative (10), defining community standards for data representation in proteomics, provide a good example to illustrate this. The original standard for proteomic experiments, MIAPE—Minimum Information About a Proteomics Experiment (11), is a Reporting Guideline first developed in 2007 to guide the capture of metadata pertaining to proteomic experiments. Over time this standard has diversified to include eight other standards, covering mass spectrometry experiments, informatics, quantification, gel electrophoresis, column chromatography and more. These extensions are standards in their own right, but are related to each other and the original MIAPE standard. We capture all this information using our Related Standards interface (see Figure 1). We also capture when these standards are implemented in a database (e.g. the PRIDE database at the EBI, see figure), so allowing users to see the level of adoption of a standard. These standards also evolve over time. We capture when a standard is replaced by a new version, or split into more than one standard. We also record when a standard is deprecated. We retain the record, but add a note explaining the deprecation and, where applicable, direct users to a new standard that is related to or that supersedes the deprecated standard.

**Figure 2:**
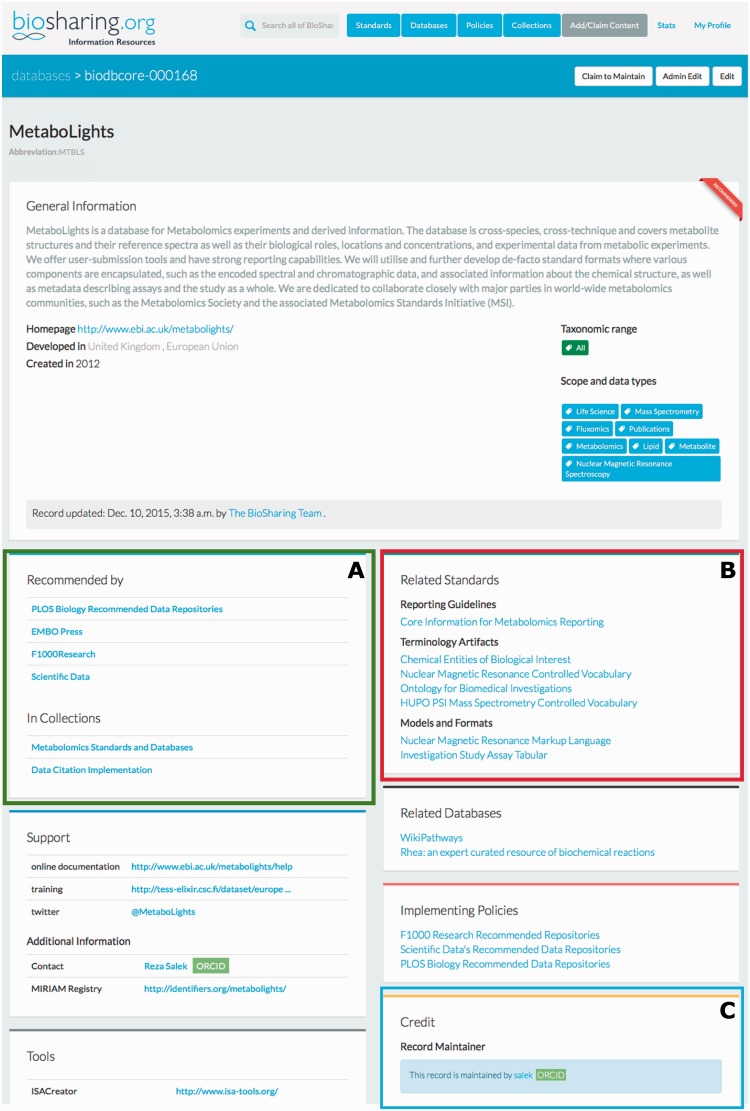
The top section of the BioSharing Metabolights Database Record. Every record on BioSharing starts with a general information section, containing a description of the resource, and details of the domains and species that the resource covers. Further, more specific details are found in the boxes beneath this section. If a resource is selected in a Collection or Recommendation, this information is provided in the ‘In Collections’ section (**A**—highlighted in green). The standards implemented in the Metabolights database are found in the Related Standards section (**B**—highlighted in red), split into the three standard subtypes. The Metabolights database record is maintained by the resource themselves. This information is found in the Credit section (**C**—highlighted in blue). Clicking on the maintainer link takes you to the profile for the maintainer, with information linked from their ORCID account, if connected. This section also contains information on the group that has developed the resource and funding information. The Metabolights database is mentioned in four collections (as of November 2015). Clicking on any of the links in this section takes the user directly to the collection.

The BioSharing Database registry contains around 700 entries, curated from the published literature and direct user input. Through collaboration with Oxford University Press, we semi-automatically curate database information from the yearly Nucleic Acids Research Database issue (from 2010 onwards). The database registry follows the BioDBcore guidelines (12), which we co-developed with the International Society for Biocuration (www.biocuration.org). The BioDBcore guidelines are a community-defined, uniform, generic description of the core attributes of a biological database. BioSharing encourages and drives database developers/biocurators to follow these guidelines when submitting or editing their records. We will use the Metabolights (13) record as an example to illustrate the registry (see Figure 2). At the top of the record, as with all records in BioSharing, there is a URL linking to the homepage of the resource, and a short free text description. Below this, there is country and taxonomy information, curated using a controlled set of tags based on the list of country names provided by the United Nations and the NCBI Taxonomy (14). The data type and scope of the resource is summarised using a community-generated set of tags (see the community curation section below for more information). Below the top section of the record, there are a number of sections relating to the support information about the resource (e.g. tutorials, help mail, FAQs), contact details, publications, and the tools and web services available from the database. The Credit section of the record details the group or organisation behind the resource, and grant or funding information. This section also contains a link to the maintainer of the record (where available). This maintainer is someone associated with the resource itself, and is able to edit and update the record directly (more information about becoming a maintainer and community curation can be found in the ‘Community curation’ section below). By collating this information into one resource, and augmenting it with links to our standard and policy registries, the BioSharing database registry is the most comprehensive catalogue of biological databases available today.

Our growing Policy registry will progressively collate data preservation, management and sharing policies from international funding agencies, regulators and journals. The registry currently records over 20 data policies and links these to both standards and databases. We expect this area of BioSharing to grow substantially in the future, in collaboration with JISC (the UK higher, further education and skills sectors’ not-for-profit organisation for digital services and solutions) and as part of an RDA/Force11 joint working group (see the ‘Community Curation’ section below for more information).

## Collections and Recommendations

Our ‘Collection’ feature allows organisations and selected users to collate records across the registries, appropriate to their field or focus. This ‘grouping’ feature generates a view of the standards, databases and policies that are particular to an organisation, project or area of interest. Some of our Collections are ‘Recommendations’. These are core groupings of databases and/or standards for a particular domain, as recommended by a community group, funder, journal publisher or indeed the BioSharing team. An example of this is the Nature Publishing Group Scientific Data repositories and standards Recommendation which collates 58 databases and 5 data standards that are considered mature and stable enough to be recommended by the journal (https://biosharing.org/recommendation/ScientificData). Records mentioned in a Recommendation are flagged with a red ‘Recommended’ ribbon. We have Recommendations from a number of other journal publishers, such as BioMed Central and PLOS as well as Collections from projects such as NIH BD2K bioCADDIE and eTRIKS (https://www.etriks.org). Creating these Collections/Recommendations not only helps publishers, funding agencies, librarians and researchers filter through the sea of standards and databases in the life sciences, but also places the selected records in context within the BioSharing ecosystem, where we display information on which standards are implemented in each database, along with other relationships and links between the resources.

## Browsing and Searching BioSharing

The whole of BioSharing, the three registries and the Collections/Recommendations, can be searched from the homepage using our main search box, or the smaller search box located in the top right hand side of every page. Clicking on our Advanced Search reveals 12 field-specific searches, such as resources with particular funders or licences, or for a specific species, country or domain. Clicking on the askBioSharing logo, to the right of the search box on the homepage, provides access both to the Recommendations and the BioSharing wizard. This wizard guides users through the data in a step-by-step manner (selecting the registry, domain and species) providing users with a short list of results tailored to their requirements. In addition to the search options, the homepage provides links to each registry and some brief statistics, alongside links to help and technical documentation, an orientation tour of BioSharing, and our community, collaborators and advisory board.

**Figure 3:**
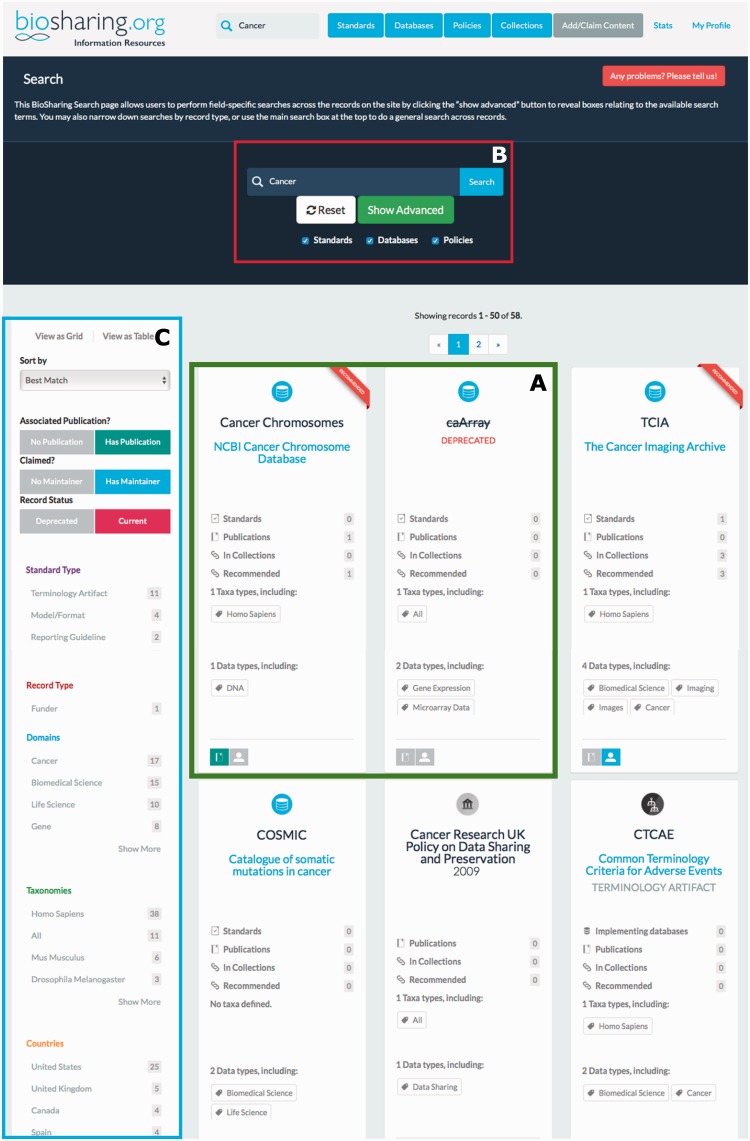
Searching BioSharing. Every search in BioSharing returns a hit list displayed as either a table or grid. In this example search, for the text ‘cancer’, 56 records have been retrieved from across the three registries and collections. Each record ‘card’ provides a snapshot of information. Note that the first search hit is recommended, and the second search hit, caArray, is a deprecated database (**A**—both highlighted in green). This search can be defined further by clicking on the advanced search option in the top search section (**B**—highlighted in red), which leads to a field-specific search. The search can be refined using the filter matrix, found down the left-hand side of the search results (**C**—highlighted in blue). This filter allows the selection of records based on a range of information. For example, whether they have a publication associated with them, if they pertain to a particular species or domain, or are associated with a particular country or funding agency.

Search hits can be viewed in a table or as ‘cards’ that display some minimal information. An example search is shown in Figure 3. The retrieved list can be ordered and filtered using the filter matrix on the left-hand side of the page. Example filters include displaying only those records that have a publication associated with them, or that are open access, or that cover a particular domain or species. Access to browse each registry or the Collections is provided by buttons at the top of every page on BioSharing.

## Community Curation

As a community effort, BioSharing offers the ability for users to add, edit and claim records and Collections. While the BioSharing team curates and integrates the descriptions of standards, databases and policies, we know that the creators and maintainers of these resources know their data best. If you are a database curator or developer, a standard maintainer, or policy advisor, you can help BioSharing by claiming and maintaining the record for your resource. If a resource is missing you can submit a new record in the appropriate registry. Why should you claim a record? First and foremost, to make your resource more discoverable to others. Second, to get credit, both for you and your group/organisation. Claiming a record is a one-off event, after which the BioSharing team will continue to link your resource(s) to others, making them even more visible. Once users have signed-up for an account, they can add or edit the record(s) for their resource via our online data entry forms. These forms, tailored to each registry type or subtype, are a mixture of optional and mandatory fields. To help users complete the forms, there is a short explanation above each field or section, detailing the type of data therein. Some fields are free text (e.g. the description field, common to all records), while others have a few restrictions (e.g. the homepage URL field must contain a valid URL). A third type of field contains a restricted list of options, accessible via a drop-down menu (e.g. the support section, containing options such as online documentation, tutorial, or contact email). Finally, a number of fields contain a semi-structured list of controlled vocabulary terms. For example, the Taxonomy and Scope and Data Types fields (present on Standard and Database records) capture the species covered using the NCBI taxonomy ontology, and the nature of the resource using controlled tags from four categories—Process, Material, DataType and Property. These tags are taken from community ontologies, such as those of the OBO Foundry, and are currently organised in a semi-structured list.

If you are a database developer or curator, you can add a section to your database website where you describe your database, following the BioDBcore guidelines, detailing (among other information) what type of data is in the database, whether it is open access, and what tools and documentation are available. We are exploring methods to automatically ingest this information into BioSharing, so making it easier for maintainers to update the metadata surrounding their resource.

BioSharing is driven by the community it serves. An international Advisory Board, with members drawn from the worlds of research, publishing, content standards, funding agencies and data resources, provides guidance and advice on our direction, user interface and curation. We also receive feedback from record maintainers and users, along with community interest. BioSharing has many users, from different backgrounds and with different needs, from the standard developer to the journal editor. Coordinating, accommodating and serving these diverse requirements is an ongoing challenge. To help address this, BioSharing, with Advisory Board members and interested members of the community, has created a joint RDA/FORCE11 BioSharing Working Group which aims to define the principles behind the links between the content standards, databases and policies in BioSharing. To further understand our user requirements, BioSharing has recently surveyed over 500 users from around the world as part of our work with the ELIXIR EXCELERATE program, to better assess their needs for and requirements from, a standards registry in the life sciences. Feedback from this survey, and through the BioSharing website, will help define our future priorites. Through this community engagement, we hope to continue to develop BioSharing as an information resource for the life sciences, building not only on the three registries but also on the links between them, in a progressive and comprehensive manner.
